# Head and neck mycetoma: Clinical findings, investigations, and predictors for recurrence of the disease in Sudan: A retrospective study

**DOI:** 10.1371/journal.pntd.0010838

**Published:** 2022-10-17

**Authors:** Alaa T. Omer, Elfatih A. Hasabo, Sara N. Bashir, Noha E. EL hag, Yousra S. Ahmed, Istabraq I. Abdelgadir, Asma A. Osman

**Affiliations:** 1 Faculty of Medicine, University of Khartoum, Khartoum, Sudan; 2 Department of Community Medicine, Faculty of Medicine, University of Khartoum, Khartoum, Sudan; Yale University School of Medicine, UNITED STATES

## Abstract

**Introduction:**

Mycetoma is a unique neglected tropical disease which is found endemic in areas known as the “mycetoma belt”. Head and neck mycetoma is a rarity and it has many devastating impacts on patients and communities. In this study, we assessed clinical findings, investigations, and predictors for recurrence of head and neck mycetoma in Sudan.

**Methodology:**

A retrospective study was conducted at Mycetoma Research Center in Khartoum between January 1999 and December 2020 for all patients with head and neck mycetoma. Data were analyzed using R software version 4.0.2.

**Results:**

We included 107 patients with head and neck mycetoma. 65.4% were young adult males from mycetoma endemic areas in Sudan, and most of them were students (33.6%). Most of patients (64.4%) had actinomycetoma. Before presenting with head and neck mycetoma, majority (75.7%) had a long duration with mycetoma, and 30.8% had a history of trauma. The commonest invaded site was the parietal region (30.8%). The lesion started gradually in most of the patients (96.3%). 53.3% of the patients had large size lesions with no sweating, regional lymph nodes involvement, or distal vein involvement. CT scan was the most accurate diagnostic tool while 8.4% of patients were diagnosed by clinical examinations only. Laboratory investigations confirmed that 24/45 (44.4%) of actinomycetoma was caused by Streptomyces somaliensis while 13/28 (46.4%) of eumycetoma was caused by Madurella mycetomatis. All patients with recurrence of head and neck mycetoma underwent surgical excision of the lesion (n = 41/41 {100%}, p < 0.001).

**Conclusion:**

In head and neck mycetoma, the most common type was actinomycetes in Sudan. Majority had a long course of mycetoma and the commonest causative organism was Streptomyces somaliensis. The treatment outcome was poor and characterized by a low cure rate.

## Author summary

Head and neck mycetoma is the rarest form of mycetoma. It has devastating impacts on patients, communities, and the health system. The study concludes that: Head and neck Mycetoma -like all mycetoma- mainly affects young adult males in rural areas of Sudan. The disease is diagnosed using numerous radiological methods and investigations alongside clinical examination. Despite the CT scan being the most accurate tool, most patients are diagnosed by clinical tests only; due to the high cost of investigations and financial issues. In this study, all patients with the recurrence of head and neck mycetoma underwent surgical excision of the lesion. The worst thing about the head and neck Mycetoma is that the treatment outcome was poor and characterized by a low cure rate. More attention in is needed regarding mycetoma globally and in Sudan, especially in Gezira and Sinnar states. Therefore, screening by history and clinical examination of Mycetomata’s lesions is mandatory for persons living in an endemic regions.

## Introduction

Mycetoma -or Madura foot- is a unique neglected tropical disease that typically affects poor communities and is characterized by causing massive deformity and disability that could be fatal for patients [1]. It is a chronic granulomatous infection of the skin, subcutaneous tissue, and bone resulting in painless firm mass with discharge that contains grains of the organism that may contribute to the delay of presentation for many patients [1–4].

Mycetoma is caused by bacteria (actinomycetoma) or fungi (eumycetoma). Actinomycetoma is more prevalent in dry areas, whereas eumycetoma is more common in rainy areas. More than 59 organisms (Fungi and bacteria) are suggested to cause this disease. However, common bacteria are: Actinomadura madurae, Nocardia brasiliensis, Streptomyces somaliensis, and Actinomadura pelletieri, while common fungi are: Madurella mycetomatis, Madurella niger, and Leptosphaeria senegalensis [5,6].

The most affected area is the foot, in addition to other sites such as the face and neck have also been reported;therefore, people who work barefooted were mainly affected, like farmers and herders. However, other occupations are at risk for having mycetoma [5,7] Mycetoma is not transmitted from person to person and without known vector or animal reservoir. It affects all age groups, and it is uncommon in children [2].

The diagnosis of mycetoma requires several tools, including the culture of grains and imaging techniques such as radiography, ultrasonography, computerized tomography, magnetic resonance image, molecular techniques -such as PCR-, serodiagnosis -such as ELISA-, and histopathological diagnosis [8–10].

Although the global burden of mycetoma is unknown, this disease is found endemic in tropical and subtropical areas in the so-called “Mycetoma belt” which includes the Bolivarian Republic of Venezuela, Chad, Ethiopia, India, Mauritania, Mexico, Senegal, Somalia, Sudan, Thailand, and Yemen. Most cases occur in the mycetoma belt, which is situated between latitudes 15° south and 30° north [11–13]. These areas have hot weather, a rainy season for three months, and a dry season for nine months which results in the growth of acacia trees [5].

Early lesions with early presentation respond well to medical and surgical treatment with a good prognosis [14,15]. In general, actinomycetoma responds to medical therapy (antibiotics), while eumycetoma requires antifungal and surgical excision [16,17].

The recurrence of the disease occurs in most patients and is common in patients with fungal infections and among those who underwent surgical excision [16–18].

A previous study in Sudan showed that more than 7000 patients received treatment for mycetoma at the mycetoma research center (MRC) despite awareness and effort in 2010 [2]. In 2014, a study on 49 patients with head and neck mycetoma at MRC reported that most of the patients were young adults, which affects the productive age [18].

In this study, we aimed to estimate the proportion of head and neck mycetoma, clinical findings, causative agents, investigations, and predictors for recurrence of head and neck mycetoma.

## Methods

### Ethics statement

Ethical approval of the study was obtained from the Technical and Ethical Review at Community Medicine Department Board in the Faculty of medicine, University of Khartoum, Khartoum, Sudan (serial number 6\2021). The study was carried out following the relevant ethical guidelines and regulations.

### Study design and duration

This was a retrospective study conducted at the Mycetoma Research Centre, University of Khartoum, Khartoum state, Sudan for all participants with head and neck mycetoma between January 1991 till December 2020.

### Study area

MRC is located in Soba, Khartoum state, Sudan, and its considered the only center in Sudan that specialized in mycetoma. The MRC was established in 1991 at Soba university hospital, University of Khartoum, Khartoum, Sudan. Since 1991, MRC provided regular follow-up for more than 9000 patients [19]. MRC is know with high-quality medical care, research about mycetoma, and excellent education. In addition, it contains a lab for the diagnosis of mycetoma, besides a special radiological and surgical department. All medications were given freely for all mycetoma patients at MRC.

### Participants

All patients with head and neck mycetoma seeking health care at MRC and have a mycetoma lesion in the head or neck in one or more sites or who have mycetoma in more than one part of the body, one of these parts must be the head or neck. These are our inclusion criteria, and there are no exclusion criteria regarding this research.

### Sampling

We enrolled all consecutive participants who met our inclusion criteria during the study period. The total number of patients with head and neck mycetoma were 107 patients.

### Diagnosis of mycetoma

The diagnosis of mycetoma was confirmed by careful interview, meticulous clinical examinations, and standard investigations. The investigations included FNAC (fine needle aspiration cytology), histopathological examination of biopsies using variable staining techniques, and grains culture in different media (take four weeks before discarded as negative) beside counter-immuno-electrophoresis.

X-ray scan was used for the affected sites with at least two views—anterior-posterior view and lateral view-. Ultrasound examination was used at the lesion to see the grains and capsule of mycetoma, and the inflammatory granuloma. Some patients used computed tomography (CT) scan and MRI which showed dot in a circle sign. Due to the financial condition of patients and the unnecessary investigations, laboratory and radiological investigations were requested for all patients.

### Data collection technique and tools

A data extraction sheet was used to collect data from files. The study included all subjects with a confirmed diagnosis of head and neck mycetoma seen in the period January 1991 to December 2020.

Due to the coronavirus disease-19 (COVID-19) pandemic, data were collected retrospectively from medical files of patients via a data extraction sheet.

The data extraction sheet contains the following information:

- Socio data: it contained age, gender, occupation, and residence of the participants.
- Clinical history section which contains the onset of the lesion, duration, pain, discharge, and associated sweating as well as the history of trauma, family history, and history of a medical problem.
- Clinical examination: it described the site and size of mycetoma, the number of affected areas, and the presence of sinus, grain, or discharge. Also, the color of the discharge and involvement of lymph node or vein.
- Methods of diagnosis: included findings of radiological investigations (X-ray, CT, MRI, or US), and laboratory investigations (cytology, histopathology, and serology).
- Final section contained the type of management, type of medications, number of previous surgery, and complications.

### Statistical analysis plan

Data were imported into Microsoft Excel for cleaning and analyzed using R software version 4.0.2. Continuous data were presented as mean ± standard deviation (SD), and categorical data were presented as numbers (percentage). Data were expressed in the form of tables. Independent t-tests; fisher’s exact test and the chi-squared test were used to find the differences between groups with and without the recurrence of head and neck mycetoma.

## Results

This study included 107 patients with confirmed head and neck mycetoma constituting 1.15% of the total MRC patients (9299) seen during the duration of the study.

This report included 49 patients from the previous study “Head and neck Mycetoma: The Mycetoma Research Center Experience” [18].

### Sociodemographic data

The age of participants in the study ranged from a minimum of 7 years to a maximum of 95 years (Range = 88 years) with a mean (SD) age of 27.7 ± 16.0 years. There were 70 patients (65.4%) in the study below 30 years. Male to female ratio is equal to 2.34 (75 males: 32 females). For occupation, 36 patients (33.6%) were students, while four (3.7%) were employers. (Table 1).

**Table 1:** Baseline characteristics and clinical history of participants with mycetoma. (n = 107).

Variables	Overall, N = 107^1^	Recurrence of the disease		p-value^2^
		Yes, N = 41^1^	No, N = 66^1^	
**Age, years**	27.7 ± 16.0	30.9 ± 17.9	25.8 ± 14.6	0.11
**Age, years (Groups)**				0.5
Less than 19 years	33 (30.8%)	10 (24.4%)	23 (34.8%)	
19 to 29 years	37 (34.6%)	14 (34.1%)	23 (34.8%)	
30 to 40 years	20 (18.7%)	8 (19.5%)	12 (18.2%)	
41 to 51 years	6 (5.6%)	4 (9.8%)	2 (3.0%)	
Equal to or more than 52 years	11 (10.3%)	5 (12.2%)	6 (9.1%)	
**Gender**				0.3
Female	32 (29.9%)	10 (24.4%)	22 (33.3%)	
Male	75 (70.1%)	31 (75.6%)	44 (66.7%)	
**Occupation of subject**				0.7
Employer	4 (3.7%)	2 (4.9%)	2 (3.0%)	
Farmer	21 (19.6%)	11 (26.8%)	10 (15.2%)	
Freelancer	23 (21.5%)	9 (22.0%)	14 (21.2%)	
Housewife	13 (12.1%)	4 (9.8%)	9 (13.6%)	
Jobless	10 (9.3%)	4 (9.8%)	6 (9.1%)	
Student	36 (33.6%)	11 (26.8%)	25 (37.9%)	
**Living state**				0.4
Gedaref state	5 (4.7%)	3 (7.3%)	2 (3.0%)	
Geziar state	26 (24.3%)	11 (26.8%)	15 (22.7%)	
Darfur state	8 (7.5%)	3 (7.3%)	5 (7.6%)	
Kassala state.	9 (8.4%)	1 (2.4%)	8 (12.1%)	
Khartoum state	12 (11.2%)	7 (17.1%)	5 (7.6%)	
Kordofan state	19 (17.8%)	5 (12.2%)	14 (21.2%)	
River Nile state	5 (4.7%)	1 (2.4%)	4 (6.1%)	
Northern state	2 (1.9%)	1 (2.4%)	1 (1.5%)	
Outside Sudan	2 (1.9%)	0 (0.0%)	2 (3.0%)	
Red Sea state	1 (0.9%)	0 (0.0%)	1 (1.5%)	
Sinnar state	9 (8.4%)	4 (9.8%)	5 (7.6%)	
White Nile state	9 (8.4%)	5 (12.2%)	4 (6.1%)	
**History of trauma (Yes)**				**0.013**
No history of trauma	66 (61.7%)	22 (53.7%)	44 (66.7%)	
Not sure	8 (7.5%)	7 (17.1%)	1 (1.5%)	
Yes	33 (30.8%)	12 (29.3%)	21 (31.8%)	
**History of medical problem (Yes)**	2 (1.9%)	1 (2.4%)	1 (1.5%)	>0.9
**Sweating around the lesion**				0.6
Absent	104 (97.2%)	39 (95.1%)	65 (98.5%)	
Present	3 (2.8%)	2 (4.9%)	1 (1.5%)	
**Family history of mycetoma (Yes)**	6 (5.6%)	4 (9.8%)	2 (3.0%)	0.2

^*1*^ Mean ± SD; n (%)

^*2*^ Two Sample t-test; Fisher’s exact test; Pearson’s Chi-squared test

Our participants resided in 3 countries: Sudan, Chad, and Somalia. Regarding Sudan, they resided in 11 states and more than 50 localities. (Table 1).

### Clinical history

The duration of the disease at presentation ranged between three months and 35 years (Ranged = 34.75 years) with a mean (SD) duration of 5.56 ± 0.61 years. In 91 patients (85%), the duration of the lesion at presentation was equal to or less than ten years. Also, 103 patients (96.3%) documented that the onset of mycetoma lesion was gradual. (Table 1).

Pain at the mycetoma site was not a frequent symptom; it was documented in 23 patients (21.5%) (Table 2). Also, 33 patients (30.8%) recalled a history of local trauma at the mycetoma site, while 74 (69.2%) patients with no history of trauma or could not recall history of trauma. (Table 1).

**Table 2:** Clinical course and duration of the disease among participants. (n = 107).

Variables	Overall, N = 107^1^	Recurrence of the disease		p-value^2^
		Yes, N = 41^1^	No, N = 66^1^	
**Onset of the disease**				0.2
Gradual	103 (96.3%)	38 (92.7%)	65 (98.5%)	
Sudden	4 (3.7%)	3 (7.3%)	1 (1.5%)	
**Duration of the disease, years**	5.6 ± 6.3	6.2 ± 7.4	5.2 ± 5.6	0.4
**Duration of the disease (Categories)**				0.2
Less than 1.5 year	26 (24.3%)	12 (29.3%)	14 (21.2%)	
1.5 to 10 years	65 (60.7%)	20 (48.8%)	45 (68.2%)	
11 to 20 years	12 (11.2%)	7 (17.1%)	5 (7.6%)	
More than 20 years	4 (3.7%)	2 (4.9%)	2 (3.0%)	
**Pain**				0.6
Painful	23 (21.5%)	10 (24.4%)	13 (19.7%)	
Painless	84 (78.5%)	31 (75.6%)	53 (80.3%)	
**Most common site of the disease (Multiple answers)**				
Ear	2 (1.9%)	0 (0.0%)	2 (3.0%)	0.5
Forehead	19 (17.8%)	7 (17.1%)	12 (18.2%)	0.9
Mandible (Jaw)	6 (5.6%)	1 (2.4%)	5 (7.6%)	0.4
Maxillary area	4 (3.7%)	2 (4.9%)	2 (3.0%)	0.6
Neck	16 (15.0%)	9 (22.0%)	7 (10.6%)	0.11
Occipital	8 (7.5%)	1 (2.4%)	7 (10.6%)	0.2
Temporal	13 (12.1%)	5 (12.2%)	8 (12.1%)	>0.9
Orbital	11 (10.3%)	3 (7.3%)	8 (12.1%)	0.5
Parietal	35 (32.7%)	15 (36.6%)	20 (30.3%)	0.5
**Presentation of patients according to the site of the disease**				0.6
Ear	2 (1.9%)	0 (0.0%)	2 (3.0%)	
Forehead	17 (15.9%)	6 (14.6%)	11 (16.7%)	
Mandible (Jaw)	6 (5.6%)	1 (2.4%)	5 (7.6%)	
Maxillary area	4 (3.7%)	2 (4.9%)	2 (3.0%)	
Neck	12 (11.2%)	7 (17.1%)	5 (7.6%)	
Neck and forehead	1 (0.9%)	1 (2.4%)	0 (0.0%)	
Neck and occipital region	2 (1.9%)	0 (0.0%)	2 (3.0%)	
Neck and temporal region	1 (0.9%)	1 (2.4%)	0 (0.0%)	
Occipital region	5 (4.7%)	1 (2.4%)	4 (6.1%)	
Orbital region	11 (10.3%)	3 (7.3%)	8 (12.1%)	
Parietal and occipital regions	1 (0.9%)	0 (0.0%)	1 (1.5%)	
Parietal region	33 (30.8%)	15 (36.6%)	18 (27.3%)	
Parietal, temporal, and forehead regions.	1 (0.9%)	0 (0.0%)	1 (1.5%)	
Temporal region	11 (10.3%)	4 (9.8%)	7 (10.6%)	
**Number of areas involved by the disease**				>0.9
One	100 (93.5%)	39 (95.1%)	61 (92.4%)	
Two	6 (5.6%)	2 (4.9%)	4 (6.1%)	
Three	1 (0.9%)	0 (0.0%)	1 (1.5%)	

^*1*^ n (%)

^*2*^ Fisher’s exact test; Pearson’s Chi-squared test

A previous history of medical illness was documented in only two patients (1.9%) and both of them were due to hypertension. Also, 101 (94.4%) patients reported no family history of mycetoma. (Table 2).

### Clinical examination

**Fig 1:**
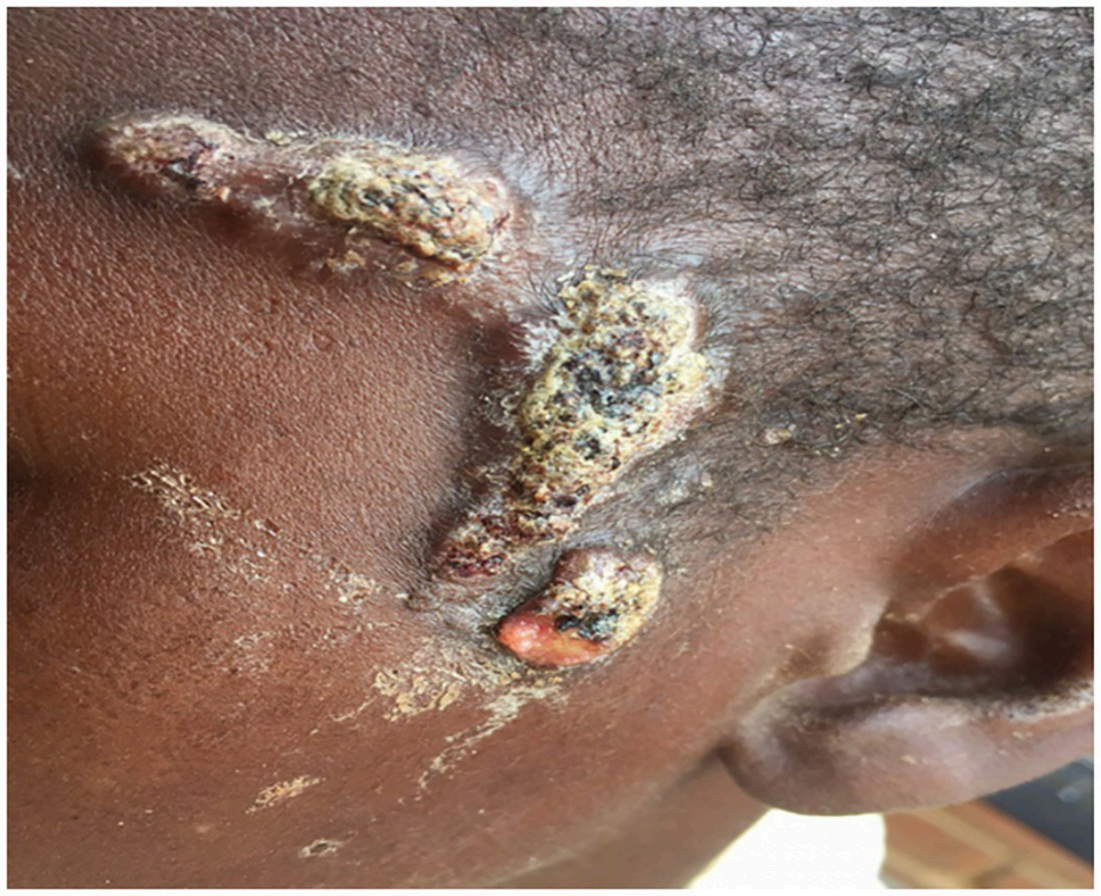
Eumycetoma in boy in the left temporal region with extension to a maxillary area with multiple sinuses and discharge.

**Fig 2:**
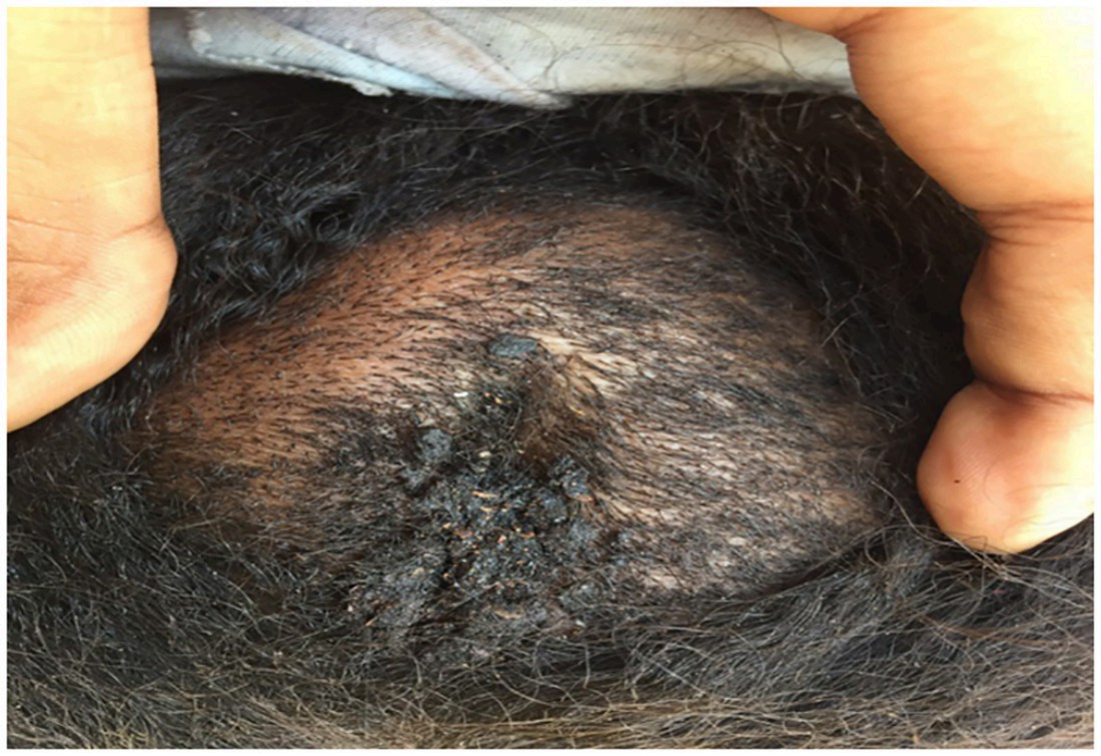
Head eumycetoma in girl in the occipital region, with intracranial involvement.

Different parts were involved in the head and neck. Only 16 patients (14.9%) had a lesion in the neck and 95 patients (88.7%) with a lesion in the head. Seven patients (6.5%) experienced a combined injury in more than one area, six of them (5.6%) had injury in two areas, and one patient (0.9%) with three involved areas. (Table 2). Figs 1 and 2 showed eumycetoma in the temporal and occipital regions.

The mycetoma lesions were classified according to their sizes into small (less than 5 cm), moderate lesions (5–10 cm), and massive lesions (>10cm). The study showed that 29 (27.1%) patients had small lesions, while 57 (53.3%) patients had massive lesions. (Table 3).

**Table 3:** Characteristics of mycetoma and lymphatic involvement in participants. (n = 107).

Variables	Overall, N = 107^1^	Recurrence of the disease		p-value^2^
		Yes, N = 41^1^	No, N = 66^1^	
**Vein involvement**				0.054
Absent	104 (97.2%)	38 (92.7%)	66 (100.0%)	
Present	3 (2.8%)	3 (7.3%)	0 (0.0%)	
**Size of the lesion, centimeter**				0.9
Less than 5	29 (27.1%)	10 (24.4%)	19 (28.8%)	
5 to 10	21 (19.6%)	8 (19.5%)	13 (19.7%)	
more than 10	57 (53.3%)	23 (56.1%)	34 (51.5%)	
**Sinuses**				0.4
Active	22 (20.6%)	9 (22.0%)	13 (19.7%)	
Active and healed	11 (10.3%)	3 (7.3%)	8 (12.1%)	
Healed	23 (21.5%)	12 (29.3%)	11 (16.7%)	
Non-active	51 (47.7%)	17 (41.5%)	34 (51.5%)	
**Lymph node involvement**				0.7
Absent	101 (94.4%)	38 (92.7%)	63 (95.5%)	
Present	6 (5.6%)	3 (7.3%)	3 (4.5%)	
**Purulent discharge from the injury**				0.2
No discharge	50 (46.7%)	16 (39.0%)	34 (51.5%)	
Purulent discharge	57 (53.3%)	25 (61.0%)	32 (48.5%)	
**Grains**				0.6
Absent	89 (83.2%)	33 (80.5%)	56 (84.8%)	
Present	18 (16.8%)	8 (19.5%)	10 (15.2%)	
**Color of discharge**				0.2
Black	16 (15.0%)	8 (19.5%)	8 (12.1%)	
Mixed	5 (4.7%)	0 (0.0%)	5 (7.6%)	
No discharge	50 (46.7%)	16 (39.0%)	34 (51.5%)	
Red	2 (1.9%)	1 (2.4%)	1 (1.5%)	
White	7 (6.5%)	2 (4.9%)	5 (7.6%)	
Yellow	27 (25.2%)	14 (34.1%)	13 (19.7%)	
**Colour of the grains**				0.3
Black	6 (5.6%)	4 (9.8%)	2 (3.0%)	
Mixed	2 (1.9%)	0 (0.0%)	2 (3.0%)	
No grains	88 (82.2%)	33 (80.5%)	55 (83.3%)	
White	3 (2.8%)	2 (4.9%)	1 (1.5%)	
Yellow	8 (7.5%)	2 (4.9%)	6 (9.1%)	

^*1*^ n (%)

^*2*^ Fisher’s exact test; Pearson’s Chi-squared test

At the presentation time, 56 patients (52.3%) had lesions with sinuses. These lesions with sinuses were active in 22 patients (20.6%), healed in 23 patients (21.5% %), and 11 patients (10.3%) had both active and healed sinuses. Local hyper-hydrosis at/and around the mycetoma lesion was detected in three patients (2.8%). Also, grains discharged from the sinuses were not detected on clinical examination in 89 patients (83.2%), while in 57 (53.3%) patients, purulent discharge was detected. Dilated tortuous veins distal to the mycetoma lesions were detected in three patients (2.8%), while regional lymph nodes enlargement was just seen in six patients (5.6%). (Table 3).

### Diagnosis

In this study, 69 patients (64.4%) had actinomycetoma, 35 (32.7%) with eumycetoma, and three patients (2.7%) highly suspected cases of mycetoma (they have a history of other fungal infections, specifically one patient for each chromoblastomycosis, Aspergilloma, and mucormycosis). (S1 Table).

Nine patients (8.4%) were diagnosed only by history and clinical examination, without laboratory or radiological investigation, and 45.7% were diagnosed using laboratory and radiological methods. Also, 43 patients (40.2%) didn’t use radiological investigations, and 25 patients (23.4%) didn’t use laboratory investigations to identify the causative agent for mycetoma. (Table 4).

**Table 4:** Diagnostic findings in patients with head and neck mycetoma. (n = 107).

Variables	N	Overall, N = 107^1^	Recurrence of the disease		p-value^2^
			Yes, N = 41^1^	No, N = 66^1^	
**Types of mycetoma**	107				0.6
Actinomycetoma		69 (64.5%)	25 (61.0%)	44 (66.7%)	
Aspiregllioma		1 (0.9%)	0 (0.0%)	1 (1.5%)	
Chromoblastomycosis		1 (0.9%)	0 (0.0%)	1 (1.5%)	
Eumycetoma		35 (32.7%)	15 (36.6%)	20 (30.3%)	
Mucormycosis		1 (0.9%)	1 (2.4%)	0 (0.0%)	
**Radiological method of diagnosis**	107				**0.022**
CT		13 (12.1%)	3 (7.3%)	10 (15.2%)	
CT and X-ray		2 (1.9%)	0 (0.0%)	2 (3.0%)	
Not done		43 (40.2%)	14 (34.1%)	29 (43.9%)	
Ultrasound		15 (14.0%)	5 (12.2%)	10 (15.2%)	
US and CT		3 (2.8%)	0 (0.0%)	3 (4.5%)	
X-ray		21 (19.6%)	15 (36.6%)	6 (9.1%)	
X-ray and ultrasound		10 (9.3%)	4 (9.8%)	6 (9.1%)	
**CT scan findings**	18				0.6
Bone destruction		1 (5.6%)	0 (0.0%)	1 (6.7%)	
Innovation of organ		6 (33.3%)	0 (0.0%)	6 (40.0%)	
No change		2 (11.1%)	0 (0.0%)	2 (13.3%)	
Soft tissue involvement		9 (50.0%)	3 (100.0%)	6 (40.0%)	
**Ultrasound findings**	28				0.3
Bone involvement		4 (14.3%)	0 (0.0%)	4 (21.1%)	
Grain and forign body		2 (7.1%)	0 (0.0%)	2 (10.5%)	
Grain are founded		17 (60.7%)	7 (77.8%)	10 (52.6%)	
No grain		4 (14.3%)	1 (11.1%)	3 (15.8%)	
Spinal compression		1 (3.6%)	1 (11.1%)	0 (0.0%)	
**X-ray findings**	33				0.5
Bone destruction		11 (33.3%)	7 (36.8%)	4 (28.6%)	
No change		11 (33.3%)	5 (26.3%)	6 (42.9%)	
Preosteam reaction		3 (9.1%)	1 (5.3%)	2 (14.3%)	
Soft tisue mass		8 (24.2%)	6 (31.6%)	2 (14.3%)	
**Methods of diagnosis**	107				0.6
Clinical and laboratory method		34 (31.8%)	11 (26.8%)	23 (34.8%)	
Clinical and Radiological method		15 (14.0%)	8 (19.5%)	7 (10.6%)	
Clinical, Radiological, and laboratory methods		49 (45.8%)	19 (46.3%)	30 (45.5%)	
History and clinical examination only		9 (8.4%)	3 (7.3%)	6 (9.1%)	
**Serology results**	10				0.3
Actinomadura pelletieri		1 (10.0%)	1 (12.5%)	0 (0.0%)	
Actinomadura madurae		1 (10.0%)	0 (0.0%)	1 (50.0%)	
All types of organisms*		1 (10.0%)	1 (12.5%)	0 (0.0%)	
Negative		3 (30.0%)	2 (25.0%)	1 (50.0%)	
Streptomyces somaliensis		4 (40.0%)	4 (50.0%)	0 (0.0%)	
**Cytology results**	29				**0.044**
Actinomadura madurae		5 (17.2%)	4 (44.4%)	1 (5.0%)	
Madurella mycetomatis		14 (48.3%)	3 (33.3%)	11 (55.0%)	
No grain is seen		2 (6.9%)	1 (11.1%)	1 (5.0%)	
Streptomyces somaliensis		8 (27.6%)	1 (11.1%)	7 (35.0%)	
**Histopathology results**	58				0.4
Actinomadura madurae		10 (17.2%)	2 (9.1%)	8 (22.2%)	
Actinomadura madurae and Actinomadura pelletieri		2 (3.4%)	1 (4.5%)	1 (2.8%)	
Actinomadura pelletieri		2 (3.4%)	1 (4.5%)	1 (2.8%)	
Madurella mycetomatis		16 (27.6%)	9 (40.9%)	7 (19.4%)	
Streptomyces somaliensis		23 (39.7%)	7 (31.8%)	16 (44.4%)	
Unknown		5 (8.6%)	2 (9.1%)	3 (8.3%)	
**laboratory investigation result**	82				0.7
Actinomadura pelletieri		2 (2.4%)	2 (6.5%)	0 (0.0%)	
Actinomadura madurae		18 (22.0%)	6 (19.4%)	12 (23.5%)	
Actinomadura madurae and Actinomadura pelletieri		2 (2.4%)	1 (3.2%)	1 (2.0%)	
All types of organisms*		1 (1.2%)	1 (3.2%)	0 (0.0%)	
Madurella mycetomatis		21 (25.6%)	8 (25.8%)	13 (25.5%)	
Negative		3 (3.7%)	1 (3.2%)	2 (3.9%)	
Streptomyces somaliensis		31 (37.8%)	11 (35.5%)	20 (39.2%)	
Unknown		4 (4.9%)	1 (3.2%)	3 (5.9%)	

^*1*^ n (%)

^*2*^ Fisher’s exact test; Pearson’s Chi-squared test

All types of organisms*: Actinomadura pelletieri, Actinomadura madurae, and Streptomyces somaliensis

At presentation, the duration of 56.5% of actinomycetoma lesions and 68.5% of eumycetoma patients ranged from 1.5 to 10 years. 28.5% of eumycetoma and 31.8% of actinomycetoma patients are diagnosed only by the laboratory method.

At presentation, 33 patients (30.8%) had a skull and cervical x-ray in at least two views. For eumycetoma and x-ray, 6/13 (46.1%) patients showed no change in x-ray, 3/13 (23%) patients show soft tissue mass, 2/13 (15.3%) show periosteal reaction and 2/13 (15.3%) show bone destruction. On the other hand, for actinomycetoma and x-ray, only 5/20 (25%) patients show no change in x-ray, and 5/20 (25%) show soft tissue mass, one (0.5%) showed a periosteal reaction, and nine (45%) showed bone destruction.

CT scan was not requested for 89 patients (83.2%). 2/18 (11.1%) showed no change, 9/18 (0.5%) showed soft tissue involvement and 6/18 (33.3%) showed organ innovations while one (5.5%) patient showed bone destruction. In this series 28 (26.1%) patients underwent US investigation and in 17/28 (60.7%) patients grains are founded, 2/28 (7.1%) showed grains and foreign body, 4/28 (14.2%) show no grain, 1/28 (3.5%) show spinal compression, and 4/28 (14.2%) only show bone involvement. (Table 4).

Laboratory investigations confirmed that 24/69 (44.4%) of actinomycetoma was caused by Streptomyces somaliensis while 13/35 (46.4%) of eumycetoma was caused by Madurella mycetomatis. (S1 Table) FNAC was performed in 29 (27.1%) patients to confirm the diagnosis while 58 (54.2%) were diagnosed using histopathology for surgical biopsies, and ten (9.3%) were diagnosed using serology. One patient with each Aspergilloma and chromoblastomycosis was diagnosed by histopathology. Aspergilloma was caused by aspergillus while chromoblastomycosis was caused by blastomycosis. Laboratory investigations showed evidence of Actinomadura madurae in 18 (22.0%) patients, M. mycetomatis in 21 (25.6%), and Streptomyces somaliensis in 31 (37.8%). During laboratory investigation, Actinomadura pelletieri -without the presence of any other causative agent- caused actinomycetoma in two patients, and more than one organism was found in three patients. (Tables 4 and S1).

### Treatment and predictors with the recurrence of head and neck mycetoma

Regarding treatment and predictors with the recurrence, 41 (38.3%) patients underwent surgical excisions for the mycetoma lesion, and this was the main predictor with the recurrence (P-value < 0.001). Only 69 (64.5%) patients used antibiotics as a type of treatment while 38 (35.5%) used antifungals. However, 41 (38.3%) patients were managed using drugs and surgery. (Table 5).

**Table 5:** Management and complications in patients with head and neck mycetoma. (n = 107).

Variables	Overall, N = 107^1^	Recurrence of the disease		p-value^2^
		Yes, N = 41^1^	No, N = 66^1^	
**Drug of treatment**				0.5
Antibiotic	69 (64.5%)	25 (61.0%)	44 (66.7%)	
Antifungal	38 (35.5%)	16 (39.0%)	22 (33.3%)	
**Type of management**				**<0.001**
Drugs and surgery	41 (38.3%)	41 (100.0%)	0 (0.0%)	
Drugs only	66 (61.7%)	0 (0.0%)	66 (100.0%)	
**Number of previous surgeries to the injury**				**<0.001**
0	66 (61.7%)	0 (0.0%)	66 (100.0%)	
1	28 (26.2%)	28 (68.3%)	0 (0.0%)	
2	5 (4.7%)	5 (12.2%)	0 (0.0%)	
3	5 (4.7%)	5 (12.2%)	0 (0.0%)	
4	2 (1.9%)	2 (4.9%)	0 (0.0%)	
5	1 (0.9%)	1 (2.4%)	0 (0.0%)	
**Complications of mycetoma**				0.4
Eye protrusion	1 (0.9%)	1 (2.4%)	0 (0.0%)	
Loss of hearing	1 (0.9%)	0 (0.0%)	1 (1.5%)	
No complications	99 (92.5%)	39 (95.1%)	60 (90.9%)	
Reached the brain	6 (5.6%)	1 (2.4%)	5 (7.6%)	

^*1*^ n (%)

^*2*^ Fisher’s exact test; Pearson’s Chi-squared test

For eumycetoma, the common antifungal agents used were ketoconazole (400–800 mg\day) and Itraconazole (400 mg daily) beside the surgical excision. The liver function is checked before and during the treatment as it may be affected by the drugs.

For actinomycetes, the first line of treatment is a combination of amikacin sulfate (15 mg\Kg) and co-trimoxazole(14 mg\kg\twice daily). In resistance cases, streptomycin sulfate (14 mg\ kg\ day) combined with co-trimoxazole (14 mg\kg\ twice daily) OR combined with rifam ((15–20 mg\kg\day). Treatment must be continued until the patient is cured.

58/69 (84%) patients with actinomycetoma need one or zero surgical excision while for eumycetoma 33/35 (94.2%) patients need one or zero surgical excision. There is no data available to determine the number of patients cured, those lost to follow up, and other outcome.

## Discussion

Our retrospective study showed that incidence of mycetoma in the head and neck regions was infrequent, and patients with confirmed head and neck mycetoma constituted 1.15% of the total MRC patients seen during the study duration. In 1986, researchers found 3.75% of cases with mycetoma involving the head and neck region, and in 1964 only 0.96% had head and neck mycetoma, while in Senegal, it was 2% and in Mexico 3% [18,20]. These findings were similar to our study and confirmed that mycetoma in head and neck was not common.

The majority of the reported patients (65.4%) were young adults with a mean age of years 27.73 + 1.55 years which is considered the typical age in mycetoma patients and this could be explained that this age group is more active and working in farms and fields where the organism is found. Our study found males were predominantly affected in and this is consistence with previous reports from Sudan [1,21,22]. The explanation for this is unclear, but it is assumed that males were predisposed to thorn prick and trauma more than females due to their work, and another assumption said that sex hormones play a role in this predominance [23]. Most affected occupations were farmers and freelancers (41.1%) and this is an important finding as the nature of their work put them in direct contact with the soil and it has been postulated that the soil harbors the causative organisms and these patients are constantly exposed to minor injuries which facilitate the traumatic subcutaneous inoculation of the organisms. Students represented 33.6% of total participants, and this may be explained that young patients were more likely to contract the disease and aware for seeking health care than other age groups. Also, 9.3% of the patients were unemployed due to prolonged illness and disability. The majority of the patients (32.7%) were from central Sudan -Gazira State and Sinnar State-, followed by Kordofan state, which constituted 17.8% of total patients. Only one patient resided in Red Sea state in eastern Sudan. This may be due to the nature of the soil in Kordofan and central Sudan, where it is suitable for the growth of thorns, not like the soil of the Red Sea state.

The clinical presentation of patients in this study was typical and in agreement with other reports [2,11]. It started gradually at the subcutaneous tissue and progressed till the affection of the deep structures. In addition, only 3.7% of the patients recorded sudden presentation. The disease’s mean duration among the affected population was quite long and the majority of the patients (85%) had mycetoma for 10 years or less, and only four patients had the disease for more than 20 years. The long duration of the disease may be explained by the painless nature of the mycetoma, the lack of health education, low socio-economic status of the affected patients, use of herbal medicine, going to “Fakkis” (traditional healer), and lack of medical and health facilities in the endemic regions [20]. Head and neck mycetoma was painless in the majority of patients (78.5%) and this may be an important contributory factor for the late presentation in most patients. However, head and neck mycetoma was painful in 21.5% of the patients and this may be due to secondary bacterial infection during infection mycetoma [13]. Regarding the history of trauma, 69.2% of the patients could not recall an injury or they didn’t have trauma, and this high percentage could be explained by forgottenness of patients; due to the long duration since time of injury or the trauma being small in size.

The clinical presentation of the site of mycetoma in our participants was not typical with previous studies (18,20). Different parts of the head and neck areas were affected. The parietal region was affected most (30.9%) in our participants. This explanation is unclear; however, this part is more prone to direct trauma and some people were carrying wood -which contains the causative organisms of mycetoma- in this part of the head. The ear was considered the least part been infected with head and neck mycetoma (1.9%).

At the time of presentation, almost half of the patients had massive lesions due to their late presentation and most of them had actinomycetoma (64.4%) which is known to be aggressive and can invade the deep structures and bone at an early disease stage [1]. Also, 56 patients (52.3%) had lesions with active sinuses, healed, or both which is considered one of the features for mycetoma lesion [18], while 51 (47.7%) patients had no sinuses. This high percentage of patients without sinuses in their lesions may be a new feature or the patient missed understanding the question -presence of sinus- during the history taking. In 16.8% of patients, grains of the causative organism was detected. Several reasons could explain this low percentage: examiner missed the grains due to the small size of it, the patient clean the lesion before the time of examination, or a small amount of discharge or the color of the discharge prevents the vision of the grains. In 53.3% of the patients, discharge was detected during the examination due to the response of the immune system to the lesion [5] Local hyper-hydrosis at or and around the mycetoma lesion was seen in three patients, and this may be a rare feature of mycetoma or it is only a discharge from the lesion. Dilated tortuous veins distal to the mycetoma lesions were detected in three patients, and this finding was not found in a previous study [18]. This could be explained by the presence of mycetoma lesion, which prevents blood flow in the veins [18], or the three patients already have a vascular problem before the mycetoma lesion. Regional lymph nodes enlargement was detected in six patients (5.6%) and this correlates with a previous study [18].

In the present study, the prevalent type was actinomycetes (64.4%) and this is in agreement with the previously reported studies [2,18]. However, the explanation for the high prevalence of mycetoma with actinomycetes remained unclear, but a previous study showed that actinomycetes might be resilient and able to survive in the extra-pedal areas more than eumycetes [2,18].

The ideal protocol for diagnosis of mycetoma is using clinical, laboratory, and radiological methods together. In our study, more than half of patients (54.3%) were diagnosed by one method only, 8.4% of the patients diagnosed only by clinical features–history and clinical examination- without laboratory or radiological investigation, and 40.1% of the patients didn’t perform radiological investigations. The reasons for not performing investigations were due to refusing of patients, low socioeconomic status of patients, and low capacity of MRC devices. Also, 19.6% of total patients were diagnosed using x-ray only and this may lead to aggravation of the disease and loss of the function of the organ; due to the limited ability of x-ray to show the damaged tissue caused by mycetoma. For actinomycetoma, 9/20 (45%) patients showed bone destruction due to its ability to survive in the extra-pedal areas more than eumycetes; therefore, it can invade deep bone [2]. Regarding ultrasound investigation, 14% of the patients did this investigation. They found grains in 60.7% of total patients, but grains and foreign body was found in 7.1% of them and the percentage nearly the same for findings grains using clinical examination.

Our study showed that 23.4% of the patients were diagnosed without laboratory investigations and this was explained by refusing to follow the doctor’s guides, or financial issues of participants. Even though Mycetoma mycetomatis caused 60% of mycetoma in Sudan, the majority of head and neck mycetoma was caused by the actinomycetes, particularly Streptomyces somaliensis, followed by Madurella mycetomatis, Actinomadura madurae and Actinomadura Pelletieri [5].

This study showed that 61.7% of the patients treated only with drug -without surgery- and this is explained by the good response of mycetoma to the drug, while 48.3% of the patients need surgery and this is explained by the patients’ dissatisfaction due to the high cost and the long treatment duration which is commonly more than one-year duration, the drug side effects and complications, the patients low socioeconomic status, the lack of health education and difficulty to reach the MRC, particularly during rainy seasons. All these reasons can contribute to the poor treatment outcome; therefore, the second line of treatment via surgery will be done for patients with mycetoma [18,24]. The study showed that most patients with actino-mycetoma (84%) need one or no surgical excision, while for eumycetoma, 94.2% of the patients need one or no surgical excision. It is well known that incomplete surgical excision performed under local anesthesia is the major factor leading to recurrence [12].

The recurrence of the disease in this study was found to be 100% among patients who underwent previous surgical excision, and this is a significant percentage (P < 0.001). Compared with a previous study, the recurrence rate among the patient who underwent the previous excision was 73.5%, which supports this study’s result [18].

Our study focused and highlighted all the characteristics of the lesion in patients with head and neck mycetoma. Also, we investigated the predictors for recurrence of head and neck mycetoma and included all head and neck mycetoma patients in Sudan from 1999 to December 2020.

However, in addition to these strengths, we faced some limitations. Firstly: our study was retrospective in design; therefore, we can’t find the mortality among these participants. Secondly, implementing the study in a single center and a low sample size may affect the generalizability of these results. Thirdly, the culture, size, and form of the grain from etiological agents were determined relying on morphological technique, which is a limitation of precise species specification.

Finally, there are insufficient numbers of patients with head and neck mycetoma and there are missing data in the records and there are different used terms for describing the size of mycetoma’ injury in records (the size is small, the size like a coffee bean, the size is 5 * 5 cm).

## Conclusion

In conclusion, mycetoma in head and neck regions is rare and associated with severe complications, a low cure rate, and a high dropout rate during follow-up. Most of the patients were young adult males and students. Majority of the patients the lesion took place in the parietal region and had a large size with no sweating, regional lymph nodes involvement, or distal vein involvement.

Regarding the investigations, the most ordered radiological investigation was X-ray while the most ordered laboratory investigation was a histopathological biopsy. The commonest type in Sudan was found to be actinomycetoma and the laboratory investigations confirmed that most actinomycetoma is caused by Streptomyces somaliensis while most eumycetoma is caused by Madurella mycetomatis. The main predictor of recurrence of the disease was found to be the surgical excision of the lesion.

More attention in is needed regarding mycetoma globally and especially in Sudan. Health education mission is recommended to educate people in endemic areas about mycetoma, its complications, and the way of transmission for mycetoma. More centers are needed in Sudan, specifically in Gezira and Sinnar states. In addition, screening and surveillance program by history and clinical examination for mycetoma lesions is recommended for those who live in endemic areas.

More funding is required in MRC to improve the quality of service for the patients and to perform all required investigations freely for mycetoma patients. Also, further follow-up study should be established to measure the mortality among theses patients.

## Supporting information

S1 Table Result of laboratory according to the type of mycetoma. (DOCX)
