# Supplementary material for: Head and neck mycetoma: Clinical findings, investigations, and predictors for recurrence of the disease in Sudan: A retrospective study
Source: PLoS Negl Trop Dis. 2022 Oct 17;16(10):e0010838. doi: 10.1371/journal.pntd.0010838 (PMC9576061; doi:10.1371/journal.pntd.0010838)
Supplement: S1 Table — (DOCX) [file pntd.0010838.s001.docx]

| **S1 Table**: Result of laboratory according to the type of mycetoma. | | | | | | | | |
| --- | --- | --- | --- | --- | --- | --- | --- | --- |
| **Variable** | **N** | **Overall**, N = 107*^1^* | **Actinomycetoma**, N = 69*^1^* | **Aspiregllioma**, N = 1*^1^* | **Chromoblastomycosis**, N = 1*^1^* | **Eumycetoma**, N = 35*^1^* | **Mucormycosis**, N = 1*^1^* | **p-value***^2^* |
| **labrotary investigation result** | 82 |  |  |  |  |  |  | **0.001** |
| Actinomadura pelletieri |  | 2 (2.4%) | 2 (3.8%) | 0 (0.0%) | 0 (0.0%) | 0 (0.0%) | 0 (NA%) |  |
| Actinomadura madurae |  | 18 (22.0%) | 14 (26.9%) | 0 (0.0%) | 0 (0.0%) | 4 (14.3%) | 0 (NA%) |  |
| Actinomadura madurae and Actinomadura pelletieri |  | 2 (2.4%) | 1 (1.9%) | 0 (0.0%) | 0 (0.0%) | 1 (3.6%) | 0 (NA%) |  |
| All types of organisms* |  | 1 (1.2%) | 0 (0.0%) | 0 (0.0%) | 0 (0.0%) | 1 (3.6%) | 0 (NA%) |  |
| Madurella mycetomatis |  | 21 (25.6%) | 8 (15.4%) | 0 (0.0%) | 0 (0.0%) | 13 (46.4%) | 0 (NA%) |  |
| Negative |  | 3 (3.7%) | 1 (1.9%) | 0 (0.0%) | 0 (0.0%) | 2 (7.1%) | 0 (NA%) |  |
| Streptomyces somaliensis |  | 31 (37.8%) | 24 (46.2%) | 0 (0.0%) | 0 (0.0%) | 7 (25.0%) | 0 (NA%) |  |
| Unknown |  | 4 (4.9%) | 2 (3.8%) | 1 (100.0%) | 1 (100.0%) | 0 (0.0%) | 0 (NA%) |  |
| All types of organisms*: Actinomadura pelletieri, Actinomadura madurae, and Streptomyces somaliensis | | | | | | | | |
